# Intussusception hospitalizations before rotavirus vaccine introduction: Retrospective data from two referral hospitals in Tamil Nadu, India

**DOI:** 10.1016/j.vaccine.2017.11.043

**Published:** 2018-12-14

**Authors:** Rajan Srinivasan, C.P. Girish Kumar, Sridevi A. Naaraayan, Susan Jehangir, Jeromie W.V. Thangaraj, S. Venkatasubramanian, Gagandeep Kang

**Affiliations:** a Division of Gastrointestinal Sciences, Christian Medical College, Vellore, India; b ICMR – National Institute of Epidemiology, Chennai, India; c Institute of Child Health and Hospital for Children, Chennai, India; d Department of Pediatric Surgery, Christian Medical College, Vellore, India

**Keywords:** Intussusception, Retrospective surveillance, Rotavirus vaccine, Children

## Abstract

**Background:**

The indigenous oral rotavirus vaccine Rotavac® was introduced into the public immunization system in India in 2016 and will be expanded in phases. This data will describe the epidemiology of intussusception in India in absence of rotavirus vaccination and will help in setting up or designing a safety monitoring system.

**Methods:**

Medical records of intussusception cases between 2013 and 2016 in two major referral hospitals in Tamil Nadu, India were reviewed, and data on clinical presentation and management and outcome were collated.

**Results:**

A total of 284 cases of intussusception were diagnosed and managed at the two centers of which 280/284 could be classified as level 1 by the Brighton criteria. Median age at presentation was 8 months (Inter Quartile Range, IQR 6–17.2) with a male to female ratio of 2.1:1. Over half (57.7%) required surgical intervention while the rest underwent non-surgical or conservative management.

**Conclusions:**

Retrospective data from referral hospitals is sufficient to classify cases of intussusception by the Brighton criteria. These baseline data will be useful for monitoring when rotavirus vaccination is introduced.

## 1 Introduction

The World Health Organization recommends introduction of rotavirus vaccine in national immunization programmes (NIPs) of countries with high under -five mortality due to acute gastroenteritis. As of January 2017, 84 countries have introduced these vaccines [1]. India introduced an indigenous rotavirus vaccine Rotavac® into the NIP in 2016 with a plan for phased expansion to the whole country [2]. This was following documentation of high burden of rotavirus associated gastroenteritis as an important cause of hospitalizations among young children in India [3], [4].

The two globally licensed RV vaccines have been documented to be associated with slight increase in risk of IS post vaccination but also outline benefits of lower morbidity and mortality due to rotavirus gastroenteritis which outweigh the risk [5]. Understanding the epidemiology of intussusception prior to vaccine introduction is important and will help with establishing surveillance for safety monitoring once vaccines are in wide use [6], [7]. In India, several studies have described intussusception in children using retrospective data, but there is considerable variability in the numbers of cases, their presentation and management in different health care facilities (Table 1). We describe pre-vaccine epidemiology and characteristics of intussusception among children less than 5 years between 2013 and 2016 in two major referral hospitals, one private non-for-profit hospital and the other the major government pediatric referral hospital in the state of Tamil Nadu, southern India.

**Table 1:** Studies describing intussusception (IS) among children in India.

Article	Year and author	Location	No. of hospitals	Study period	Confirmed IS cases	Presentation n (%)	Management n (%)
Intussusception hospitalizations before rotavirus vaccine introduction: retrospective data from two referral hospitals in Tamil Nadu, India	Srinivasan R et al. [Present study]	Chennai, Vellore	2	2013–2016	284	Abdominal Mass- 134 (47.2); Blood on Rectal Examination- 85 (29.9); Acute Abdominal Distension-64 (22.5); Lethargy- 36 (12.7); Pallor- 14 (4.9); Rectal Mass- 6 (2.1); Shock- 6 (2.1); Intestinal Prolapse- 2 (0.7)	Non-Surgical- 89 (31.3) Surgical- 129 (45.4) Both- 35 (12.3) Conservative- 28 (9.9)

Intussusception in Children Aged Less than Five years [14]	Mehendale S et al. 2016	Chennai	8	2012–2013	201	Vomiting- 142 (69.6); intermittent cry- 106 (52); blood in Stool- 89 (43.6); diarrhea-73 (35.8); fever-57 (27.9); abdominal pain-55 (27); irritability- 42 (20); bilious vomiting 40 (19.6); red currant jelly stool- 39 (19.1); abdominal mass- 121(60); rectal mass- 5 (2)	Surgery- 78 (38.8); Non-surgical reduction- 122 (60.6); conservative - 3 (1)

Intussusception in southern India: Comparison of retrospective analysis and active surveillance [13]	Jehangir S et al. 2014	Vellore	1	2011–2013	Active surveillance- 16	Abdominal distention-2 (12.5); vomiting- 14(87.5)	Non-surgical reduction-7 (43); Conservative-9 (56)
				January 2010 -August 2013	Retrospective surveillance- 59	Abdominal distention- 11 (18.6); vomiting- 38 (64.4); red currant jelly stool-55 (93.2); abdominal pain-28 (47.5); lethargy- 19 (32.2)	Surgery- 31 (53); Non-surgical reduction- 28 (47)

Active surveillance for intussusception in a phase III efficacy trial of an oral monovalent rotavirus vaccine in India [4]	John J et al. 2014	Vellore	1	2010–2013	11	Blood in stool- 11 (1 0 0); Vomiting- 2 (18.2); Abdominal mass- 2 (18.2)	Non-surgical reduction-11

Retrospective surveillance for intussusception in children aged less than five years at two tertiary care centers in India [19]	Singh JV et al. 2014	Lucknow, Manipal	2	2007–2012	187	Recurrent vomiting- (51.3); Abdominal pain (47%); Blood in stool (18.7%); Abdominal distension (12.3%); Excessive crying (13.4%); Fever (6.4%)	Surgery- 132 (71%); Non-surgical reduction- 48 cases (25.66%); conservative- 5 (2.6)

Retrospective Surveillance for Intussusception in Children Aged Less than Five Years in a South Indian Tertiary-care Hospital [12]	Bhowmick K et al. 2009	Vellore	1	2001–2004	31	Vomiting- 26 (83.9); Abdominal pain- 23 (74.2); Abdominal mass- 19 (61.3%); red currant jelly stool- 13 (41.9);	Surgery- 21 (67.7); Non-surgical reduction - 10 (32.2)

Population-Based Incidence of Intussusception and a Case-Control Study to Examine the Association of Intussusception with Natural Rotavirus Infection among Indian Children [20]	Bahl R et al. 2009	New Delhi	1	2000–2002	47	Excessive cry: 29 (61.7%) Bloody/currant jelly stools: 36 (76.6%); Abdominal/rectal mass 10 (21.3%); Vomiting-32 (68.1%); Abdominal distention-33 (70.2%); Diarrhea in previous 2 weeks -10 (21.3%)	Not available

Ultrasound guided hydrostatic reduction of intussusception in children by saline enema: our experience [21]	Nayak D and Jagdish S 2008	Pondicherry	1	2000–2007	102	Not available	Non-surgical reduction-83 (81.3); Surgery- 19 (18.6)

Intussusception in southern Indian children: lack of association with diarrheal disease and oral polio vaccine immunization [22]	Raman T et al. 2003 (CMC)	Vellore	1	1991–2000	165	Bloody stools- 12 (9.2); Diarrhea- 31 (11%)	Non-surgical reduction- 26 (18.9); Surgery-96 (59)

Non-ischemic intussusception in childhood [23]	Shekhawat NS et al. 1992	Jaipur	1	1966–1990	Data available-31 (total number-230)	Diarrhea (no duration): 12 (40)	Non-surgical reduction: 4; Surgery: 27

## 2 Methods

### 2.1 Study area and participating hospitals

This retrospective hospital-based analysis reviewed cases of intussusception documented in the medical records from September 2013 to October 2016, at two major referral hospitals in the state of Tamil Nadu in south India. The Institute of Child Health and Hospital for Children (ICH) is an 837-bedded government pediatric referral hospital performing more than 14000 surgeries annually in Chennai, the state capital. The Christian Medical College Hospital (CMC) is a 2800 bedded not-for- profit private referral hospital located in Vellore district performing over 2400 pediatric surgeries annually.

### 2.2 Case definition, retrospective data collection and analysis

Children <60 months of age with a diagnosis of intussusception in hospital records were eligible for inclusion in the analysis and were categorized using the criteria developed by the Brighton Collaboration Working Group [8]. Data were collected by study physicians from hospital treatment and discharge records to identify children diagnosed with intussusception and abstract clinical characteristics and management details using case report forms. The median (with IQR) was calculated for continuous variables and tested by Median test (SPSS 17.0) whereas proportions and frequency tables were used to summarize categorical variables. The studies were approved by the institutional review boards of the two institutions.

## 3 Results

**Fig. 1:**
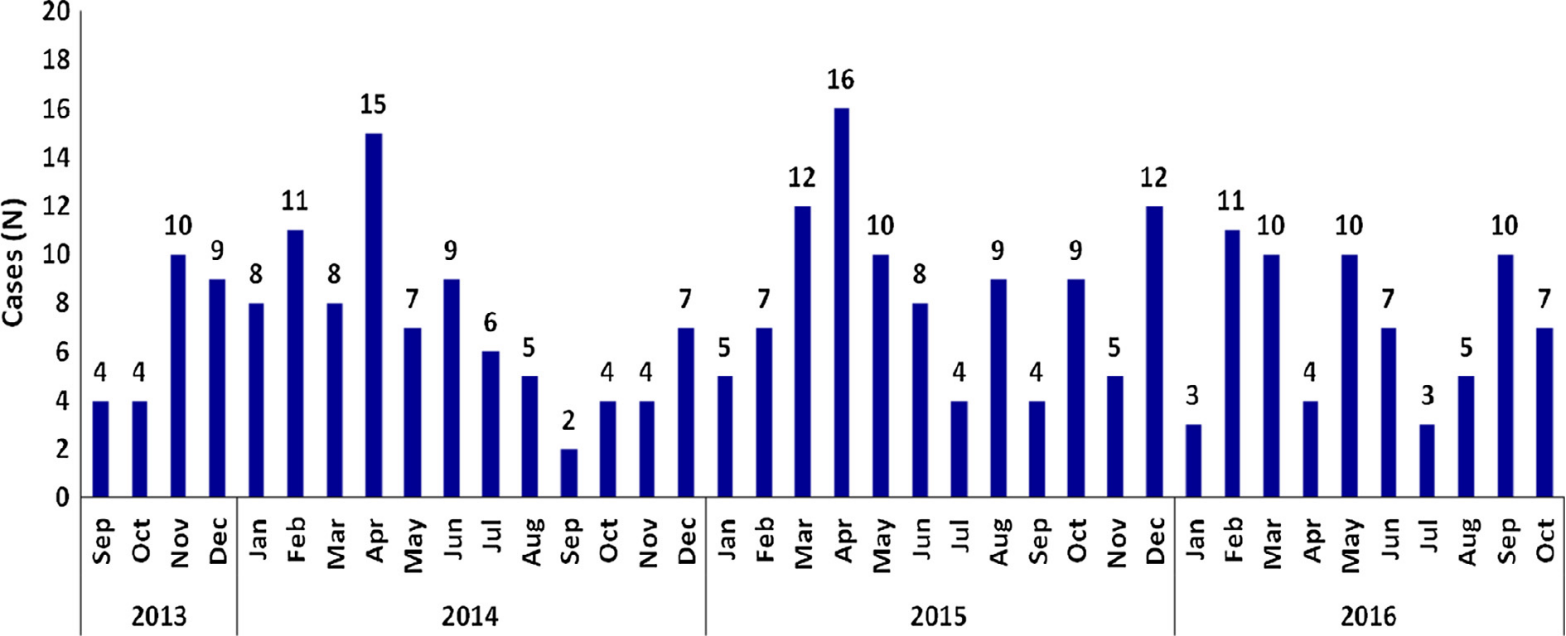
Monthly distribution of intussusception cases in children aged <60 months in Tamil Nadu.

**Fig. 2:**
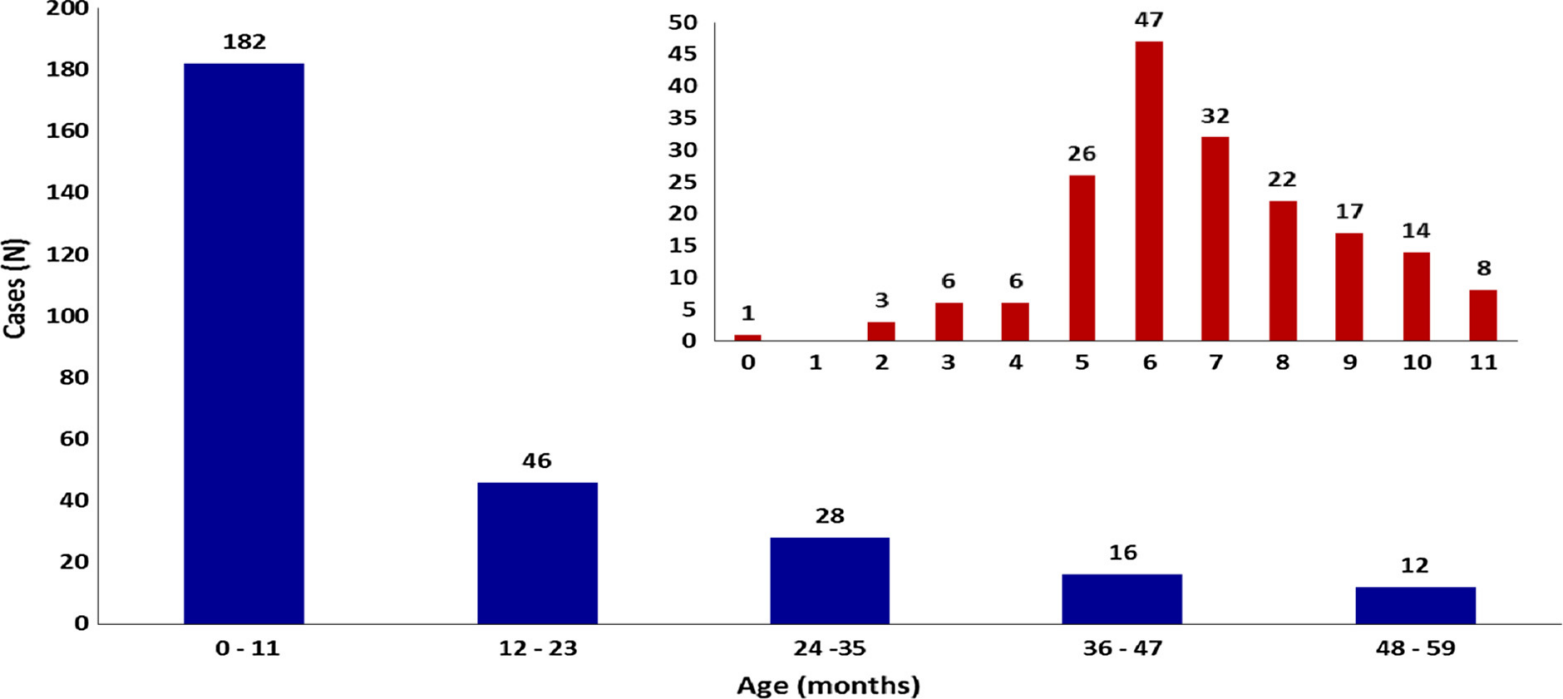
Age-group distribution of intussusception associated hospitalizations in Tamil Nadu.

Between September 2013 and October 2016, a total of 284 children aged less than 5 years, diagnosed to have intussusception were admitted to the two hospitals (Table 2, Fig. 1). The male- female ratio was 2.1:1 and the median age at presentation for males was 8 months (Inter Quartile Range, IQR 6–14.2) and for females was 9.3 months (IQR 6–18.6). Overall, the median age was 8 months (IQR 6–17.2) with 49.3% (140/284) in the age group of 6–11 months and 80.3% (228/284) below two years of age (Fig. 2).

**Table 2:** Demographic and clinical characteristics of children <5 years of age with intussusception at two referral hospitals in Tamil Nadu.

		ICH (N = 207)		CMCH (N = 77)		Total (N = 284)		p value$
Variable	Levels	N	%	N	%	N	%	
Age (m)	0–2	3	1.4	1	1.3	4	1.4	.924
	3–5	25	12.1	13	16.9	38	13.4	.290
	6–11	111	53.6	29	37.7	140	49.3	**.017**
	12–23	30	14.5	16	20.8	46	16.2	.201
	24–35	23	11.1	5	6.5	28	9.9	.246
	36–47	8	3.9	8	10.4	16	5.6	**.034**
	48–60	7	3.4	5	6.5	12	4.2	.247

Gender	Male	141	68.1	52	67.5	193	68.0	.925
	Female	66	31.9	25	32.5	91	32.0	.925

Clinical Symptoms	Vomiting	154	74.4	61	79.2	215	75.7	.399
	Intermittent Cry	131	63.3	31	40.3	162	57.0	**.000**
	Passage Blood Stained Stool	113	54.6	43	55.8	156	54.9	.850
	Abdominal Pain	105	50.7	44	57.1	149	52.5	.336
	Irritability	94	45.4	17	22.1	111	39.1	**.000**
	Diarrhea	53	25.6	25	32.5	78	27.5	.249
	Passage Blood per Rectum	49	23.7	31	40.3	80	28.2	**.006**
	Bile Stained Vomiting	48	23.2	6	7.8	54	19.0	**.003**
	Fever	35	16.9	21	27.3	56	19.7	.051
	Shortness of Breath	1	0.5	0	0.0	1	0.4	–

Clinical Signs	Abdominal Mass	106	51.2	28	36.4	134	47.2	**.026**
	Blood Rectal Examination	85	41.1	0	0.0	85	29.9	–
	Acute Abdominal Distension	46	22.2	18	23.4	64	22.5	.836
	Lethargy	25	12.1	11	14.3	36	12.7	.619
	Pallor	11	5.3	3	3.9	14	4.9	.624
	Rectal Mass	6	2.9	0	0.0	6	2.1	–
	Shock	4	1.9	2	2.6	6	2.1	.729
	Intestinal Prolapse	2	1.0	0	0.0	2	0.7	–

Diagnosis	Radiology	65	31.4	77	100.0	142	50.0	**–**
	Radiology + Surgery**	126	60.9	–	–	126	44.4	**–**
	Surgery	15	7.2	–	–	15	5.3	**–**
	Clinical	1	0.5	–	–	1	0.4	**–**

Treatment	Non-Surgical	55	26.6	34	44.2	89	31.3	**.005**
	Surgical	123	59.4	6	7.8	129	45.4	**.000**
	Both	18	8.7	17	22.1	35	12.3	**.002**
	Conservative	8	3.9	20	26.0	28	9.9	**.000**
	Others***	3	5.3	–	–	3	1.1	–

Outcome#	Recovered	198	98.5	77	100.0	275	98.9	**–**
	Died	3	1.5	–	–	3	1.1	**–**
Brighton Criteria	Level 1 (Definite)	203	98.1	57	74	260	91.5	**.000**
	Level 2 (Probable)	4	1.9	20	25.9	24	8.5	**.000**

Note:

** X-ray, USG, USG guided reduction, etc.

*** Includes 3 LAMA from ICH.

# 5 case (LAMA) + 1 case (data missing) at ICH.

$ p values <0.05 shown in bold.

The median time from the onset of symptoms to presentation at CMC was 1 day (IQR 0–2) and at ICH it was 2 days (IQR 1–3). Overall, the median time to presentation for surgical cases (2 days (IQR1-3) was longer than for children who had non-surgical or conservative management (1 day (IQR 0–2; p = .003). The most frequent symptoms observed were vomiting (75.7%; 215/284), intermittent crying (57%; 162/284), passage of blood stained stool (54.9%; 156/284) and abdominal pain (52.5%; 149/284). The proportion of children with abdominal mass (p = .026), bile stained vomitus (p = .003) intermittent crying (p = .000) or irritability (p = .000) was significantly more in children admitted at ICH whereas passage of blood per rectum (p = .006) was significantly more at CMC (Table 2). The classical triad of abdominal pain, vomiting and blood in stools was similar in both hospitals and seen in 19% (54/284) of cases and commonest site of intussusception was ileocolic (78.9%; 183/232).

A majority (123, 59.4%) of children at ICH underwent surgery with 8.7% requiring surgery following unsuccessful non-surgical interventions. Children who were successfully managed with non-surgical reduction had either saline (n = 54) or barium (n = 1) reduction.

At CMC, 44.2% (34/77) were managed non-surgically (Barium enema-8, 10%; Saline reduction -6, 8%, Pneumatic reduction- 18, 23%; Combination [barium + saline/pneumatic] 2, 3%). Six (7.8%) children underwent surgical reduction while 17 (22%) had surgery following unsuccessful non-surgical interventions and 20 (26%) were managed conservatively.

Overall, 45.4% (129/284) cases were managed surgically, 31.3% (89/284) non-surgically, while 9.9% (28/284) were managed by conservative management. Of the 124 cases who initially underwent non-surgical procedures, 28% (35/124) required surgical management following failure of such procedures. In comparison to CMC, most of the surgical interventions were performed at ICH and the difference was statistically significant (Table 2). Similarly, the proportion of cases that were managed with non-surgical or conservative measures in the two hospitals was significantly different. All (98.9%, 281/284) but three cases recovered. The three deaths were reported from ICH, of which two had been referred from another hospital. All three underwent surgical resection either at ICH or another hospital but died due to septic shock. The median length of hospital stay for intussusception cases in ICH was 4 days (IQR 3–6) which was significantly more (p = .000) than in CMC where it was 1 day (IQR 0–3).

Based on the Brighton diagnostic criteria, 91.5% (260/284) cases of intussusception had a definitive diagnosis (Level I). The remaining twenty-four cases (8.5%; 24/284) were probable cases (Level II). Among the probable cases, twenty-two patients had transient intussusception which had resolved before intervention (when re-checked in the pre-intervention ultrasound). In one case with a previous history of intussusception, X-ray findings were abnormal and underwent conservative management whereas another case left against medical advice prior to ultrasonography.

## 4 Discussion

World Health Organization recommends monitoring the risk of intussusception for countries introducing rotavirus vaccine [10], [11]. While pre-licensure studies of Rotavac® rotavirus vaccine have not shown an increased risk of intussusception, it is important that children receiving Rotavac® are monitored for risk of intussusception [9]. The purpose of the present study was to retrospectively examine intussusception data at two major referral hospitals in state of Tamil Nadu and gain learning that will be useful in establishing a prospective surveillance system to monitor intussusception after rotavirus vaccine introduction.

The number of intussusception cases seen in the present analysis is consistent with the case burden seen at the respective centers during preceding years (Table 1) [12], [13], [14]. A third (32.04; 91/284) of the children with intussusception were infants aged 3–6 months of age, which overlaps with the 3^rd^ dose of the rotavirus vaccine schedule. While the Rotavac® package insert recommends vaccine till 8 months of age, administration of the rotavirus vaccine until one year of age is permitted in the national immunization programme [15]. However, a delay in the age of vaccine administration will potentially shift the risk of vaccine associated intussusception to the maximum age-associated background risk period (median age of 8 months) as seen in our study. Understanding the age overlap of this high background level of intussusceptions and the rotavirus vaccination schedule in India is crucial in interpreting intussusception data post-vaccine introduction. There were more intussusception cases reported in certain months of each year but no seasonality was noticed. The male–female ratio of 2.1:1, though higher than observed in previous studies in the same locations, was consistent with global reports [15], [16], [17].

The classical triad of abdominal pain, vomiting and blood in stools was seen in only 19% of cases which was lower than proportion reported from other parts of India [18]. Longer median time of presentation was seen among cases at ICH that required surgical reduction and this underscores the need for timely referral to hospital. Delay in intussusception management either due to delay in seeking medical attention or time-consuming referral process through primary or secondary to tertiary care centers (like ICH) in the public health system may have potentially influenced the severity and duration of illness of such children before arrival at ICH while patients may have sought private tertiary care (CMC) directly in the early stages of intussusception. The length of hospital stay was longer at ICH as most intussusception cases underwent surgical intervention. This possibly could be due to late referral and consequent worsening of the intussusception necessitating surgical intervention. Early diagnosis and referral of children suspected with intussusception directly to tertiary pediatric centers with facilities to manage intussusception should therefore be made an integral component of trainings conducted as part of the rotavirus vaccine introduction efforts.

Information on rotavirus vaccination history was unavailable in the retrospective review of medical records; therefore, it is important that health care providers who manage intussusception are made aware of the importance to collect and record details of intussusception and vaccine history. Details on onset of intussusception and vaccine exposure will help in estimating risk of intussusception following Rotavac® or other rotavirus vaccines.

The study had limitations in that it was done with retrospective data, and these hospitals being referral centers have children referred from all over the state as well as neighboring states and this information cannot be used to calculate a rate of intussusception. The study though, provides information on intussusception load in pre-vaccine introduction scenarios and provides important insights into the epidemiology of intussusception among Indian children in different geographic and clinical settings. Role of other potential risk factors like presence of adenovirus and rotavirus infection in the causation of intussusception could not be studied as part of this retrospective analysis but should be part of future prospective studies. Therefore, a functional surveillance system to identify intussusception occurring among vaccine recipients should be an integral component of post-rotavirus vaccine introduction surveillance for adverse event following immunization at health facilities in public as well as private settings.

## Disclaimers

None.

## Declaration of competing interest

None declared.

## Conflicts of interest

No authors have declared a conflict of interest.
