# Supplementary material for: Intussusception hospitalizations before rotavirus vaccine introduction: Retrospective data from two referral hospitals in Tamil Nadu, India
Source: Vaccine. 2018 Dec 14;36(51):7820–5. doi: 10.1016/j.vaccine.2017.11.043 (PMC6290388; doi:10.1016/j.vaccine.2017.11.043)
Supplement: Supplementary data 1 [file mmc1.docx]

**Table for Fig.1: Monthly distribution of intussusception cases in children aged <60 months in Tamil Nadu**

| **Year** | **Month** | **Cases [N]** |
| --- | --- | --- |
| **2013** | Sep | 4 |
|  | Oct | 4 |
|  | Nov | 10 |
|  | Dec | 9 |
| **2014** | Jan | 8 |
|  | Feb | 11 |
|  | Mar | 8 |
|  | Apr | 15 |
|  | May | 7 |
|  | Jun | 9 |
|  | Jul | 6 |
|  | Aug | 5 |
|  | Sep | 2 |
|  | Oct | 4 |
|  | Nov | 4 |
|  | Dec | 7 |
| **2015** | Jan | 5 |
|  | Feb | 7 |
|  | Mar | 12 |
|  | Apr | 16 |
|  | May | 10 |
|  | Jun | 8 |
|  | Jul | 4 |
|  | Aug | 9 |
|  | Sep | 4 |
|  | Oct | 9 |
|  | Nov | 5 |
|  | Dec | 12 |
| **2016** | Jan | 3 |
|  | Feb | 11 |
|  | Mar | 10 |
|  | Apr | 4 |
|  | May | 10 |
|  | Jun | 7 |
|  | Jul | 3 |
|  | Aug | 5 |
|  | Sep | 10 |
|  | Oct | 7 |

**Table for Fig.2: Age-group distribution of intussusception associated hospitalizations in Tamil Nadu**

| **Age (Months)** | **Cases [N]** |
| --- | --- |
| **0 - 2** | 4 |
| **3 - 5** | 38 |
| **6 - 11** | 140 |
|  |  |
| **Age (Months)** | **Cases [N]** |
| **0 - 11** | 182 |
| **12 - 23** | 46 |
| **24 -35** | 28 |
| **36 - 47** | 16 |
| **48 - 59** | 12 |
